# Occurrence and Characterization of Microplastics in Gilthead Seabream (*Sparus aurata*) Reared in the Sardinian Coastal Environments, Italy: Implications for Consumer Exposure

**DOI:** 10.3390/foods15142479

**Published:** 2026-07-13

**Authors:** Rita Melillo, Roberto Lonis, Giuseppa Lorenzoni, Gabriella Piras, Nicola Cogoni, Simona Cau, Sara Salza, Tiziana Tedde, Riccardo Bazzardi, Monica Molotzu, Giuseppe Esposito, Bruna Vodret, Domenico Meloni, Sebastiano Virgilio

**Affiliations:** 1 Veterinary Public Health Institute of Sardinia, Complex Structure of Food Hygiene, Via Duca degli Abruzzi 8, 07100 Sassari, Italy; rita.melillo@izs-sardegna.it (R.M.); pina.lorenzoni@izs-sardegna.it (G.L.); gabriella.piras@izs-sardegna.it (G.P.); simona.cau@izs-sardegna.it (S.C.); sara.salza@izs-sardegna.it (S.S.); tiziana.tedde@izs-sardegna.it (T.T.); riccardo.bazzardi@izs-sardegna.it (R.B.); monica.molotzu@izs-sardegna.it (M.M.); bruna.vodret@izs-sardegna.it (B.V.); sebastiano.virgilio@izs-sardegna.it (S.V.); 2 ARPAS—Regional Agency for Environmental Protection of Sardinia, Via Rockfeller 58/60, 07100 Sassari, Italy; rlonis@arpa.sardegna.it (R.L.); ncogoni@arpa.sardegna.it (N.C.); 3 Veterinary Public Health Institute of Piedmont, Liguria and Valle d’Aosta, Via Bologna 148, 10154 Torino, Italy; giuseppe.esposito@izsto.it; 4 Department of Veterinary Medicine, University of Sassari, Via Vienna 2, 07100 Sassari, Italy

**Keywords:** aquaculture, microplastics, μ-FTIR

## Abstract

The objective of the present study was the determination of microplastics in Gilthead Seabream (*Sparus aurata*) reared in Italy and the assessment of consumer exposure. A total of sixty specimens were collected from four aquaculture facilities (A–D) located along the Sardinian coastal environment (Italy) and selected according to the principles of the risk assessment. From each Gilthead Seabream specimen, samples of gastro-intestinal tract (GI) and muscle (M) were obtained and digested using KOH 10% (1:10). The isolated microplastics were examined under the Stereomicroscope and characterized by Fourier transform infrared microscopy (μ-FTIR). Microplastics were identified in 25% of the samples. Positive fish showed plastic polymers in GI (22% and M (8%) respectively. The contamination was very limited, with a mean abundance of microplastics of 0.3 ± 0.56 and 1.2 ± 0.41 per fish and positive fish, respectively. Polypropylene, mainly blue, red and green in color, was the most detected polymer (72%). Consumers are exposed to plastic polymers via fish consumption, but so far, the implications for consumer exposure have not yet been quantified and are still being studied. The results of the present study represent the first contribution to the assessment of consumer exposure associated with the consumption of Gilthead Seabream (*Sparus aurata*) reared in Sardinian coastal environments, Italy.

## 1. Introduction

The expanded application and production of microplastics has led to their environmental discharge, causing ubiquitous contamination of natural ecosystems worldwide [1], as well as in several animal species [2]. Microplastics may be ingested and cause many negative effects in aquatic animals, including commercially important fishes such as the Gilthead Seabream (*Sparus aurata*) [3,4,5]. The most commonly used plastic polymers in the European Union (EU) are low- and high-density polyethylene (PE), polypropylene (PP), polystyrene (PS), poly vinyl-chloride (PVC), poly (ethylene-terephthalate) (PET) and polyurethane (PU) [6,7].

Aquatic marine plastic litter originated mainly from terrestrial sources [8] and, once in the sea, through photodegradation or thermal degradation, can change density and can be reduced to smaller and smaller fragments, giving rise to microplastics that can be ingested by several aquatic animals such as bivalve molluscs, crustaceans and fish [9,10,11]. The introduction of microplastics in fish can occur directly or indirectly [12,13,14,15,16], and then they can be accumulated in the intestinal tract [17], in the liver [18], or in the brain and eyes [12,19,20]. Several negative effects of microplastics in fish have been described, including general chronic inflammation and oxidative stress, stress response and behavioral changes [21,22]. The exposure of fish to plastic polymers is also a public health concern because they can translocate into consumable muscles [13,19,23,24]. The possible risk to human health caused by the presence of plastic polymers is still being studied: chronic exposure is of great concern because of the cumulative effect that could occur [25].

The presence of microplastics in fish has been widely described, including several economically relevant aquaculture species [26,27,28,29,30,31,32,33,34,35,36,37]. European aquaculture is going through a standstill period, and after two years of growth, the value of EU aquaculture production decreased in 2023, reaching 1.04 million tons with a total value of EUR 4.76 billion [38]. In 2024, the Italian aquaculture production reached 51,000 tons of twenty different fish species with a total value of EUR 287 million. Italy led EU trout production with 28,700 tons and a total value of EUR 115 million. The Gilthead Seabream is the main marine fish aquacultured in Italy, with a production of 9900 tons and a total value of EUR 82,100 [39]. Many Gilthead Seabream aquaculture farms are placed near anthropized areas, well known hotspots for marine plastic litter contamination [40]. Moreover, plastic polymers are widely used in Gilthead Seabream aquaculture facilities, and this species is susceptible to their ingestion from the surrounding rearing environment. In particular, tanks are made up of fiber-reinforced plastic (FRP) and high-density polyethylene (HDPE), and pipes used for aeration and water provision purposes are made up of PVC. Through alteration processes such as photodegradation or thermal degradation, they can change density and can break down into smaller fragments, giving rise to secondary microplastics [32,41,42,43,44,45].

Several studies have shown that Gilthead Seabream is a good biomonitoring species for aquaculture research and for microplastics pollution in the Mediterranean Sea [46,47,48]. Natural feeding of Gilthead Seabream includes bivalve molluscs, crustaceans and small fish, and aquacultured specimens are fed with fish feeds produced from the gastro-intestinal tracts of other fish. This has been reported to be a possible source of microplastics [49,50]. However, feeding with synthetic food generally occurs in Gilthead Seabreams weighing more than 10 mg [51]. The Mediterranean Sea is among the marine environments with the greatest accumulation of marine plastic litter due to its semi-closed structure [52,53]; the limited flow of surface waters; a densely populated coastline; and the intensity of human activities such as fishing, navigation, tourism and industry [54]. In particular, an average concentration of 0.116 fragments/m^2^ of surface area was found, up to a maximum of over 0.36 fragments/m^2^ off the Island of Elba [55]. Similar results have been found in studies conducted in Corsica and Sardinia [56]. As far as the Sea of Sardinia, the concentrations of microplastics would be variable, with generally medium-high densities. It is assumed that the variability found may be due to turbulence phenomena and the wind that insists on Sardinia [56]. At the same time, the Mediterranean Sea is a region of intense aquaculture production and the Gilthead Seabream is a top fish predator of wide interest in Mediterranean aquaculture. This species is poorly investigated for the effects that microplastics may cause, and the few available studies focused on PE [3,4,5].

The objectives of the present study were (a) to evaluate the prevalence of microplastics in samples of Gilthead Seabream (*Sparus aurata*) reared in the Sardinian coastal environments (Italy); (b) to characterize the identified microplastics through the use of Fourier transform infrared microscopy (μ-FTIR); and (c) to contribute to the assessment of consumer exposure associated with the consumption of fishery products.

## 2. Materials and Methods

### 2.1. Collection, Transport, and Storage of Samples

From November 2022 to October 2023, a total of sixty aquacultured Gilthead Seabream (*Sparus aurata*) samples were collected using a random sampling procedure from four aquaculture facilities coded A, B, C and D located along the Sardinian coastal environment (Italy). Four specimens were taken from each farm in autumn, spring, and summer quarters, and four specimens from three farms (B-C-D) in winter. It was not possible to collect samples from farm A in winter, due to internal problems at the farm. In order to protect the identity of the aquaculture facilities, the exact location of each facility remains undisclosed. Several risk factors were taken into consideration to assess the possible exposure of Gilthead Seabreams to microplastics: (a) presence of industrial settlements with large-scale use of plastic products and a high risk of discharging the raw materials of plastic production; (b) presence of large urban centers with high touristic appeal with widespread use of plastic products and poor management by consumers; (c) presence of seaports receiving a high number of plastic discharges through ballast water, ship traffic, and other commercial activities. The four aquaculture facilities (A–D) were selected based on the presence of these risk factors. The selected specimens of Gilthead Seabream with a minimum size of 20 cm (EC Reg. 1967/2006) were tagged with the appropriate progressive number and the code of the aquaculture facility (A–D) and individually wrapped in aluminum foil. The fish were sent straight under refrigeration to the laboratories of the Veterinary Public Health Institute of Sardinia in Sassari (Italy) and were frozen at −18 °C for further examination. The specimens were handled carefully to minimize physical damage during transportation.

### 2.2. Assessment of Biometric Characteristics of Gilthead Seabream (Sparus aurata)

For each sample, the total length (cm) and the weight of the fish expressed in grams were recorded in a dedicated form.

### 2.3. Sample Preparation

All the samples were thawed in a cleaned metal tray overnight just before being processed. The preparation of the samples for the digestion of biotic material included preliminary steps of precise fish dissection and tissue degradation. The Gilthead Seabreams were first rinsed with distilled water to remove skin impurities such as particles or debris and placed on metal dissection trays. From each Gilthead Seabream specimen, two samples were obtained: gastro-intestinal tract (GI) and muscle (M). The digestion process was conducted according to previous studies [57]. The GI was completely removed and placed inside a 250 mL glass flask with a glass cap, previously weighed empty. The flask was then reweighed to obtain the weight of the sample by subtraction. Subsequently, the skin was removed and 10 g of M were taken from multiple points to have a representative sample. The test samples for each matrix (GI and M) were digested using 10 mL of 10% KOH for each gram of product (1:10) and incubated at 40 °C for 48 h. The water and solutions used for the KOH 10% (*w*/*v*) digestion were filtered before use, and their contamination was assessed through the preparation of a procedural blank consisting of 10% KOH without GI and M matrix and a field blank consisting of a filter placed on a glass plate under a laminar flow hood during the processing stages in order to monitor the presence of microplastics in the environment. The total number of microplastics observed in the procedural and field blanks was subtracted from the total number of microplastics in the samples [58]. In order to eliminate any presence of particles and residual organic material larger than 500 μm, the digested solutions of the GI and M samples were subjected to a preliminary sieving with a metal sieve with a 500 μm mesh. The residues were still examined to check for the possible presence of microplastics and the obtained suspensions were neutralized before filtration using 1 M citric acid in order to limit the corrosion of silicon (Si) filters analyzed by μ-FTIR in the following stages. All the samples were filtered through polycarbonate filters with a porosity of 10 μm and 47–50 mm in diameter (Merck, Darmstadt, Germany) by a vacuum pump (Thermo Savant VLP200 Valu Pump, Thermo Fisher Scientific, Waltham, MA, USA). The labeled filters were placed in glass Petri dishes to prevent contamination and reserved for identification and quantification of microplastics.

### 2.4. Identification and Quantification of Microplastics

The filters were visually examined by an Olympus IX 73 stereo microscope (Olympus, Shinjuku, Tokyo, Japan) with magnifications from 4× to 100×, and microplastics were identified based on their morphological features (shape, color, size). Subsequently, polycarbonate filters were washed with distilled water and filtered through Si filters with a porosity of 1 μm in diameter (Thermo Fisher Scientific, USA) using a vacuum pump (Thermo Savant VLP200 Valu Pump, Thermo Fisher Scientific, USA). Si filters were placed in glass Petri dishes and left to dry at room temperature for 12–24 h. Subsequently, they were sent to the laboratories of the Regional Agency for Environmental Protection of Sardinia in Sassari to characterize the microplastics by Fourier transform infrared microscopy (μ-FTIR).

### 2.5. Characterization of Microplastics by Fourier Transform Infrared Microscopy (μ-FTIR)

The microplastics were characterized by Fourier transform infrared microscopy (μ-FTIR) with a Nicolet iN10 (Thermo Fisher Scientific, USA) as previously described [57]. The infrared signal was detected in transmission mode, exploiting the brightness of Infrared Synchrotron Radiation with a spatial resolution down to 10–20 μm. The spectral detection ranged from 700 to 4000 cm^−1^, and the spectral resolution was down to 4 cm^−1^ with 64 co-scans for each measurement. Collected spectra were compared to reference spectra of Thermo Fisher Scientific libraries (e.g., Microplastics reflection; Hummel Polymer Sample and HR Sprouse Polymers by Transmission). In order to exclude laboratory contamination and confirm the different microplastics’ similarities between the major peaks and standard spectra, a minimum match of 50% was set.

### 2.6. Cross-Contamination Avoidance and Quality Control

During the handling and processing of the samples, strict measures were carried out to mitigate cross-contamination with airborne microplastics or those eventually present in the solvents used [59]. Therefore, in order to minimize eventual contamination, operators wore only cotton clothing and nitrile gloves. All the equipment and work surfaces were thoroughly cleaned before use, and all the preparation steps were carried out under a laminar flow hood. In each sampling session, the contamination level was checked by placing a clean filter throughout the duration of the experiment to evaluate any synthetic fibers suspended in the air. All the materials (flasks, knives, and tweezers) contained no plastic components and were rinsed with demineralized water before use.

### 2.7. Statistical Analysis

The differences between the biometric characteristics (length and weight) of the samples in relation to the aquaculture facility (A–D) were compared and analyzed with a one-way ANOVA (https://statpages.info/anova1sm.html, accessed on 29 June 2026). Moreover, a multiple pairwise comparison between the means of groups through a Tukey HSD (honestly significant differences) post hoc test was carried out (https://statpages.info/anova1sm.html, accessed on 29 June 2026). The results were considered statistically significant when *p*-value < 0.05. Spearman correlation analysis (https://www.socscistatistics.com/tests/spearman/calculator, accessed on 29 June 2026) with *p*-value 0.05 was applied to investigate the relationship between biometric characteristics and microplastics ingestion rate.

## 3. Results and Discussion

### 3.1. Assessment of Biometric Characteristics of Gilthead Seabream (Sparus aurata)

Table 1 shows biometric characteristics (weight and length) of the Gilthead Seabreams from the different aquaculture facilities. A significant (*p* < 0.05) difference was reported for both biometric characteristics between the four aquaculture facilities. The length and weight of the specimens were affected by the season, except for farm A, where they remained almost constant. In farms B and C, the subjects showed a similar trend, with a decrease in length and weight in spring and an increase in summer. In farm D, a decrease in the biometric characteristics was observed since spring. The subjects with greater weight and length came from farms B and C, while the subjects with the lowest weight and length came from farm A.

### 3.2. Identification, Quantification and Characterization of Microplastics

**Figure 1:**
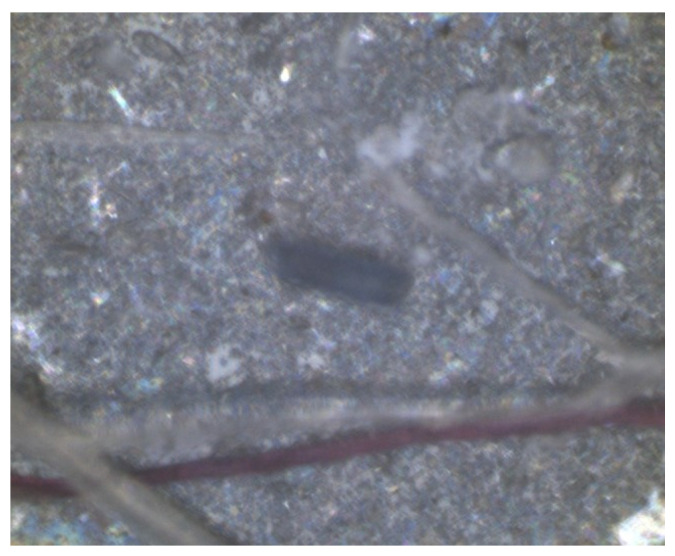
Blue polypropylene (PP) fragment (μ-FTIR image) detected in the GI sample of C 1 specimen.

**Figure 2:**
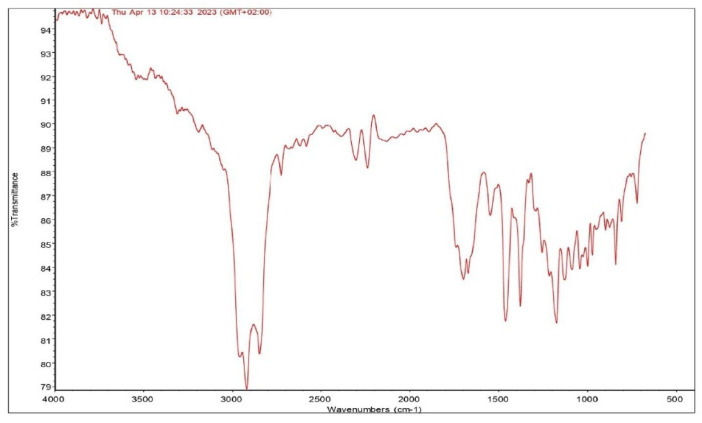
Blue polypropylene (PP) fragment (μ-FTIR spectrum) detected in the GI sample of C 1 specimen (spectrum range 700–4000 cm^−1^; spectral resolution 4 cm^−1^).

**Figure 3:**
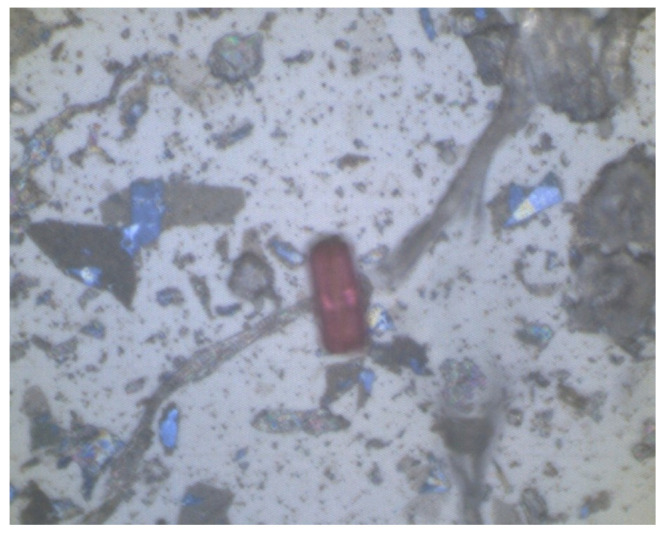
Red polypropylene (PP) fragment (μ-FTIR image) detected in the M sample of C 1 specimen.

**Figure 4:**
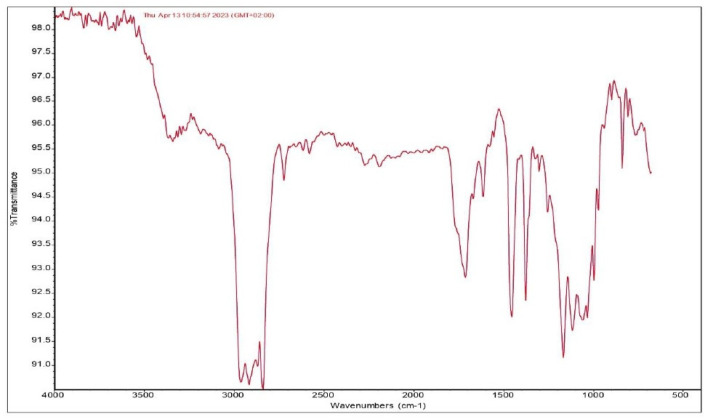
Red polypropylene (PP) fragment (μ-FTIR spectrum) detected in the M sample of C 1 specimen (spectrum range 700–4000 cm^−1^; spectral resolution 4 cm^−1^).

The presence of microplastics was highlighted in 25% of the Gilthead Seabreams (Table 1). Positive fish showed plastic polymers in GI (22%) and M (8%) respectively. The contamination was very limited, with mean abundances of microplastics of 0.3 ± 0.56 and 1.2 ± 0.41 per fish and positive fish respectively. The results of this study are in agreement with previous studies carried out on farmed Gilthead Seabreams in Europe [35,60]. The same authors reported greater prevalence of microplastics in the GI of Gilthead Seabreams farmed in Turkey (50%), Italy and Croatia (66%). In our study, microplastics frequency in farmed fish was similar to that reported in their wild counterparts. Occurrence in the GI of Gilthead Seabream was reported as 27% and 31% from the northeastern Mediterranean Sea, respectively [61]. Only one specimen (C 1) showed the simultaneous presence of microplastics (Table 2) in the GI (Figure 1 and Figure 2) and M (Figure 3 and Figure 4), both identified as polypropylene (PP) elements but of different color and appearance. PP was the most detected polymer (72%). Previous studies carried out in the Mediterranean Sea highlighted that PP is among the most frequent polymers because it is widely used in the plastics industry [7,57], and it is one of the most commonly used plastic materials in the production of fishing equipment and in aquaculture activities [62]. In general, the microplastics were all less than 0.5 mm in size and predominantly blue, red and green fragments (Table 2), supporting the hypothesis that Gilthead Seabreams ingest these plastic polymers unconsciously as they confuse them with their food [60,63]. The farms where most microplastics were detected (B and C) were those from which the heavier Gilthead Seabream originated (Table 1); however, there was no statistical correlation (*p* > 0.05) between biometric characteristics and microplastics ingestion rate.

#### 3.2.1. Aquaculture Facility A

**Figure 5:**
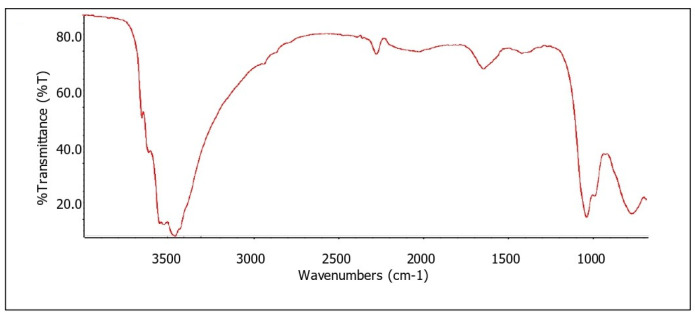
μ-FTIR spectrum of a cellophane fragment detected in the GI sample of A 10 specimen (spectrum range 700–4000 cm^−1^; spectral resolution 4 cm^−1^).

**Figure 6:**
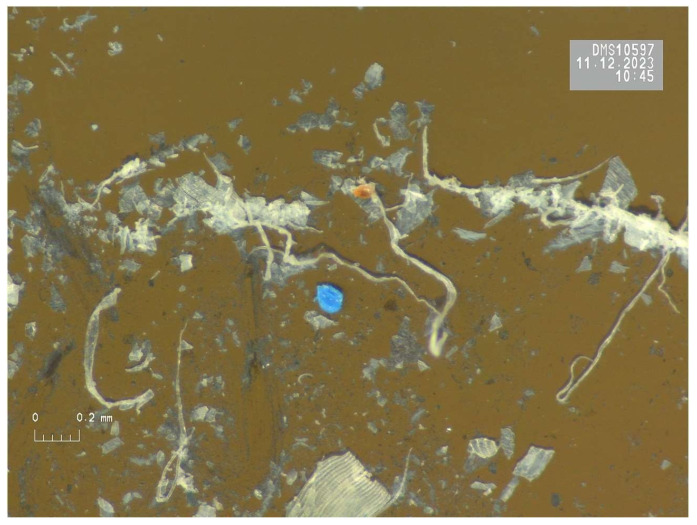
Blue phenolic resin fragment (μ-FTIR image) detected in the M sample of A 5 specimen.

**Figure 7:**
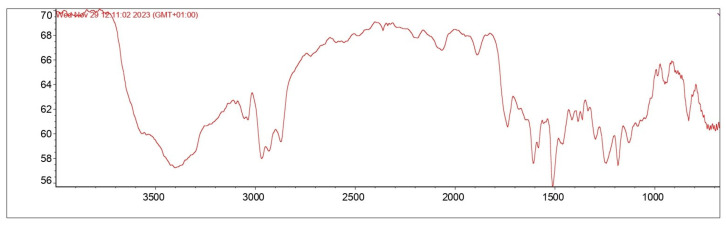
Blue phenolic resin fragment (μ-FTIR spectrum) detected in the M sample of A 5 specimen (spectrum range 700–4000 cm^−1^; spectral resolution 4 cm^−1^).

The results (Table 1 and Table 2) highlighted the presence of a provisional fragment of cellophane (Figure 5) with a relatively low library correspondence of 49% in GI of a Gilthead Seabream (A 10) sampled in autumn and a fragment of blue phenolic resin with a match of 61.62% in M of a Gilthead Seabream (A 5) sampled in summer (Figure 6 and Figure 7).

#### 3.2.2. Aquaculture Facility B

The presence of microplastics in the GI samples (B 2, B 5, B 6, B 8, B 9, B 16) was pointed out in all the considered seasons. These were nine elements of different colors but of the same chemical nature: they were blue, red and green PP fragments with a match of 68.36%, 64.57% and 65.61% respectively (Table 1 and Table 2).

#### 3.2.3. Aquaculture Facility C

**Figure 8:**
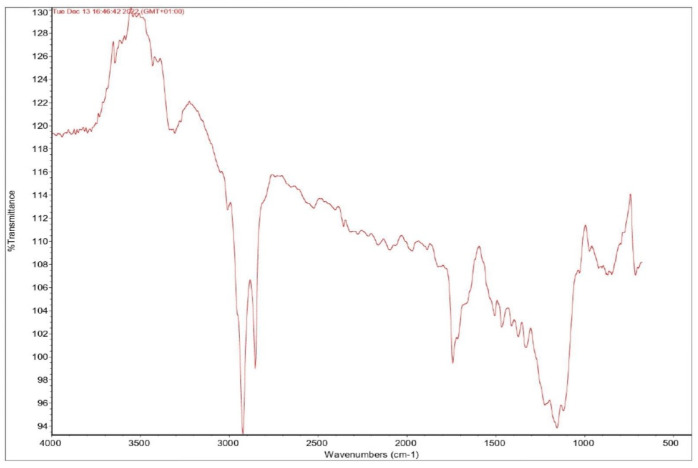
μ-FTIR spectrum of a polyethylene (PE) fragment detected in the GI sample of C 15 specimen (spectrum range 700–4000 cm^−1^; spectral resolution 4 cm^−1^).

PP fragments were detected in the GI (blue) and M (red) samples of a Gilthead Seabream (C 1) sampled in winter, with a match of 75.42% and 84.83% respectively (Figure 1, Figure 2, Figure 3 and Figure 4). The presence of a PET purple filament was pointed out in a GI sample of a Gilthead Seabream (C 13) sampled in autumn, with a match of 51.66%. In the same season, a blue PE fragment was found in a GI sample from a different Gilthead Seabream (C 15), with a match of 69.34% (Figure 8). Green PP fragments with a match of 60.11 were found in M samples of two specimens (C 10 and C 11) sampled in summer (Table 1 and Table 2).

#### 3.2.4. Aquaculture Facility D

**Figure 9:**
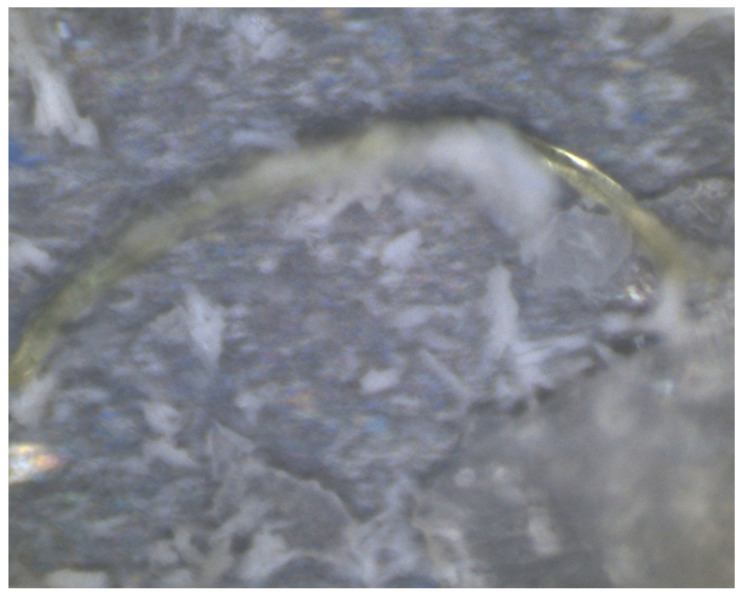
Green Nylon fiber (μ-FTIR image) detected in the GI sample of D 9 specimen and μ-FTIR (central image) and related spectrum.

**Figure 10:**
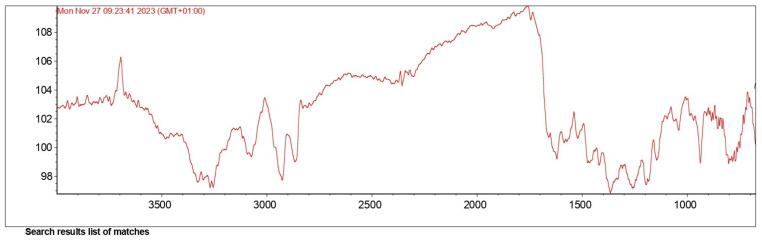
Green Nylon fiber (μ-FTIR spectrum) detected in the GI sample of D 9 specimen (spectrum range 700–4000 cm^−1^; spectral resolution 4 cm^−1^).

**Figure 11:**
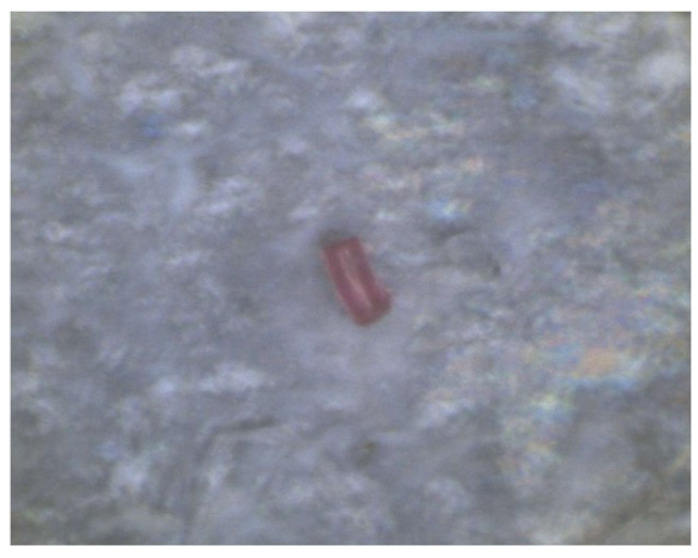
Red polypropylene (PP) fragment (μ-FTIR image) detected in the M sample of D 13 specimen.

**Figure 12:**
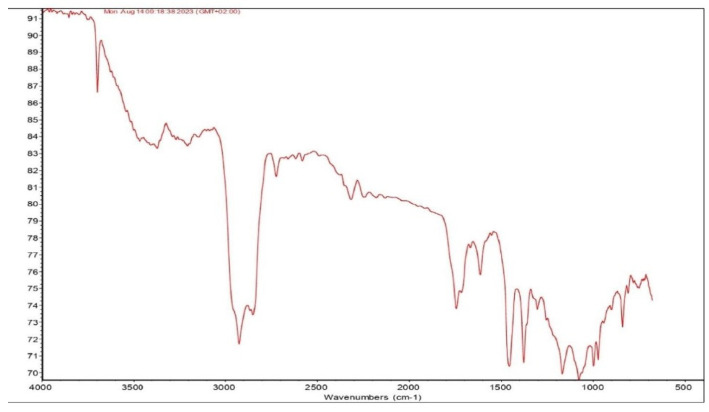
Red polypropylene (PP) fragment (μ-FTIR spectrum) detected in the M sample of D 13 specimen (spectrum range 700–4000 cm^−1^; spectral resolution 4 cm^−1^).

The results (Table 1 and Table 2) highlighted a substantial absence of microplastics in autumn, winter and spring. A nylon fiber was found in a GI sample of a Gilthead Seabream (D 9) sampled in summer with a match of 59.11% (Figure 9 and Figure 10). In the same season, a red PP fragment was found in an M sample of a different Gilthead Seabream (D 13) with a match of 79.72% (Figure 11 and Figure 12).

## 4. Conclusions

This is the first study to evaluate the prevalence of microplastics in samples of Gilthead Seabream (*Sparus aurata*) reared in the Sardinian coastal environment (Italy), underscoring the low presence of microplastics in their tissues. Plastic polymers were found in 25% of the specimens, more frequently in GI samples (22%). Only 8% of the muscle samples were positive, and contamination levels were extremely low. The μ-FTIR characterization of microplastics confirmed PP as the most detected (72%) because it is one of the most widely used in the production of fishing equipment and in aquaculture activities and can be easily ingested by aquatic organisms. Fish are usually eviscerated before consumption; however, humans should be potentially exposed to PP, but so far, the consequences have not yet been quantified. This study represents the first contribution to the assessment of the consumer exposure associated with the consumption of Gilthead Seabream (*Sparus aurata*) aquacultured in Sardinian coastal environments.

## Figures and Tables

**Table 1:** Morphology (mean ± standard deviation) of Gilthead Seabreams with microplastics abundance and frequency of occurrence.

Aquaculture Facility	Number of Fish	Total Length (cm)	Total Weight (g)	GI		M		Mean Number of Microplastics per Fish	Mean Number of Microplastics per Positive Fish	Frequency of Occurrence (%)
				n°	%	n°	%			
A	12	26.03 ± 0.25	299.26 ± 31.37	1	8	1	8	0.16 ± 0.38	1 ± 0	16
B	16	31.02 ± 1.55	502.1 ± 76.23	8	50	0	0	0.5 ± 0.73	1.33 ± 0.51	37
C	16	31.4 ± 1.90	554.57 ± 113.83	3	19	3	19	0.37 ± 0.61	1.2 ± 0.34	32
D	16	30.6 ± 2.16	481.95 ± 58.29	1	7	1	7	0.12 ± 0.34	1 ± 0	16
Total	60	-	-	13	22	5	8	0.3 ± 0.56	1.2 ± 0.41	25
Statistical analysis		*p* < 0.05	*p* < 0.05					*p* > 0.05	*p* > 0.05	

**Table 2:** Identification and characterization of microplastics collected in Gilthead Seabreams.

Aquaculture Facility	Identification of Positive Fish	Sample		Identification			Characterization (μ-FTIR)	Percentage of Matching
			Number	Size	Shape	Color		
A	5	M	1	<0.5 mm	fragment	blue	phenolic resin	61.62%
	10	GI	1		fragment	white	cellophane (provisional)	49%
B	2	GI	1	<0.5 mm	fragment	blue	polypropylene (PP)	68.36%
	5	GI	1		fragment	green	polypropylene (PP)	65.61%
	6	GI	1		fragment	green	polypropylene (PP)	
	8	GI	2		fragment	green	polypropylene (PP)	
	9	GI	2		fragment	green	polypropylene (PP)	
	16	GI	1		fragment	red	polypropylene (PP)	64.57%
C	1	GI	1	<0.5 mm	fragment	blue	polypropylene (PP)	75.42%
		M	1		fragment	red	polypropylene (PP)	84.83%
	10	M	1		fragment	green	polypropylene (PP)	60.11%
	11	M	1		fragment	green	polypropylene (PP)	
	13	GI	1		fragment	purple	poly (ethylene-terephthalate) (PET)	51.66%
	15	GI	1		fragment	blue	polyethylene (PE)	69.34%
D	9	GI	1	<0.5 mm	fiber	green	nylon	59.11%
	13	M	1		fragment	red	polypropylene (PP)	79.72%

## Data Availability

The data presented in this study are available upon request from the corresponding author due to privacy reasons and to protect the identity of the aquacultures involved in the study.
